# Genome-Wide Association Analysis of Aluminum Tolerance in Cultivated and Tibetan Wild Barley

**DOI:** 10.1371/journal.pone.0069776

**Published:** 2013-07-26

**Authors:** Shengguan Cai, Dezhi Wu, Zahra Jabeen, Yuqing Huang, Yechang Huang, Guoping Zhang

**Affiliations:** Agronomy Department, Key Laboratory of Crop Germplasm Resource of Zhejiang Province, Zhejiang University, Hangzhou, China; New Jersey Institute of Technology, United States of America

## Abstract

Tibetan wild barley (*Hordeum vulgare L. ssp. spontaneum*), originated and grown in harsh enviroment in Tibet, is well-known for its rich germpalsm with high tolerance to abiotic stresses. However, the genetic variation and genes involved in Al tolerance are not totally known for the wild barley. In this study, a genome-wide association analysis (GWAS) was performed by using four root parameters related with Al tolerance and 469 DArT markers on 7 chromosomes within or across 110 Tibetan wild accessions and 56 cultivated cultivars. Population structure and cluster analysis revealed that a wide genetic diversity was present in Tibetan wild barley. Linkage disequilibrium (LD) decayed more rapidly in Tibetan wild barley (9.30 cM) than cultivated barley (11.52 cM), indicating that GWAS may provide higher resolution in the Tibetan group. Two novel Tibetan group-specific loci, bpb-9458 and bpb-8524 were identified, which were associated with relative longest root growth (RLRG), located at 2H and 7H on barely genome, and could explain 12.9% and 9.7% of the phenotypic variation, respectively. Moreover, a common locus bpb-6949, localized 0.8 cM away from a candidate gene *HvMATE*, was detected in both wild and cultivated barleys, and showed significant association with total root growth (TRG). The present study highlights that Tibetan wild barley could provide elite germplasm novel genes for barley Al-tolerant improvement.

## Introduction

Aluminium (Al^3+^) toxicity is considered as a major factor limiting crop production on acid soils [1], [2]. Al^3+^ rapidly inhibits plant root growth, thus preventing uptake of water and nutrients [2]. The initial and visual symptom of Al toxicity is the inhibition of root elongation and development of lateral roots.

There has been no commonly-recoginized standard criterion for evaluating plant Al tolerance. Many researches have used relative root growth (RRG) to estimate Al tolerance [3]–[7]. However, a weak correlation (R^2^  = 0.17) was found between rice Al tolerance based on RRG of the longest root and RRG of the total root system [8]. In addition, an absolute root elongation was also used for Al tolerance evaluation in short-term Al stress experiments [9]–[12]. It was reported that utilization of different root index in QTL or genome-wide association analysis could be helpful to identify novel loci [13]. To make the association analysis more efficient, accurate determination of root parameters is crucial and essential. Famoso *et al* [8] developed a novel and high-throughput Al tolerance phenotyping platform, which consists of a root imaging system and root computer program. So far, this technology has been successfully applied in many research projects [14]–[15].

The Al tolerance machanism of cereals, such as wheat, barley, rice, maize and sorghum, has been extensively studied [4], [16]–[21]. The most common mechanism is secretion of organic acids, which may chelate Al in the rizosphere, thus preventing Al being absorbed by roots [22]. However, it has been reported that Al resistance in maize cannot be fully explained by root organic acid release [23]. Thus, multiple mechanisms for Al tolerance may exist in plants. Degenhardt *et al*. [24] reported that a decrease of Al^3+^ activity is caused by root-mediated increase in rizosphere pH in an Arabidopsis mutant. Furthermore, a recent research reported that a T-DNA insertional mutant of *XTH31*, a gene controlling xyloglucan endohydrolase (XEH) and xyloglucan endotransglucosylase (XET) activities, gains the function of Al resistance by modulating cell wall xyloglucan content and Al binding capacity in Arabidopsis [25]. In short, the underlying mechanism of Al tolerance is complex and still not totally understood.

Altough barley is considered as one of the most sensitive species to Al toxicity among cereal crops, there is still a wide variation in Al tolerance among cultivars or genotypes [7]. So it is meaningful to investigate the underlying mechanism of barley Al tolerance. It was widely reported that the aluminum tolerance of barley was controlled by a dominant loci on chromosome 4H. Furukawa *et al* [4] and Wang *et al* [26] identified the same candidate gene *HvMATE* (*HvAACT1*) in *cv*. Murasakimochi and Dayton respectively, which is responsible for Al-induced citrate secretion and located on the long arm of chromosome 4H. However, Gruber *et al* [27], [28] reported that over-expression of *HvALMT1*, which encodes an anion channel protein to facilitate organic anion transport, confers enhanced Al tolerance. Moreover, multigenic control of barley aluminum tolerance was reported in a few QTL studies [29], [30]. Hence, barley Al tolerance is a complex trait, which may be quantitatively inherited. On the other hand, QTL analysis has the limitation that only the alleles segregated between the parents of DH or RIL population could be identified. Hence the genetic diversity of tolerant genotypes used in QTL analysis was limited. Compared with QTL mapping, GWA can always detect more alleles in one experiment. Because natural populations, especially Tibetan wild barley accessions used in the present study, possess wider genetic variation and may contain richer tolerant alleles in comparison with a DH population.

During the extended period of domestication, especially the modern breeding and intensified cultivation, genetic diversity of cultivated barley declined gradually and rare alleles disappeared. Barren genetic background became the bottleneck of breakthrough for breeding. The Tibet plateau is considered as one of the centers of cultivated barley domestication [31]–[33]. The Tibetan wild barley has been proved to be rich in genetic diversity of stress adaptation or tolerance [34]–[35]. Due to harsh environments in Tibet, special resistant mechanisms may be developed and some excellent alleles may be formed in Tibetan wild barley. In fact, there is clear evidence that Tibetan wild barley contains elite alleles of *HKT1, HKT2* [36] and *HvCBF4* [37], conferring enhanced salinity tolerance.

In the present study, four root parameters were precisely determined by using a root morphology analysis system, which is modified according to Famoso *et al* [8], consisting of a root scanning system WinRHIZO and root image analysis software RootReader2D. The relationships between these root parameters were analyzed. Subsequently, population structure was analyzed in whole accessions, and linkage disequilibrium (LD) decays of cultivated and Tibetan wild barleys were determined and compared. Finally, genome-wide association analysis (GWAS) was conducted by using the four root parameters and 469 DArT markers within or across the cultivated barley and Tibetan wild accessions.

## Materials and Methods

### Plant materials and growth conditions

A total of 110 Tibetan wild barley accessions and 56 cultivated cultivars were used for Al tolerance identification and association mapping. All wild barleys were kindly provided by professor Sun of Huazhong Agricultural University, China [36], [37], and 56 cultivated barleys are widely planted in the different areas of China, but no one was from Tibet. Barley seeds were soaked in deionized water for 6 h at 20°C in the dark and then germinated on moist filter paper in an incubator (20/14°C, day/night) for 2 days. Then, nine seedlings (about 2 cm root length) were selected for both control and treatment, scanned by WinRHIZO [38], a root-measuring system, and images were acquired for precise analysis of root length. After scanning, seedlings were placed on the net cups floating on a 0.2 mM CaCl_2_ solution, pH 4.3, in 50 ml centrifugal tubes as control. For Al stress treatment, seedlings were exposed to a 0.2 mM CaCl_2_ solution, pH 4.3, containing 5 μM AlCl_3_. The roots were imaged for both control and Al^3+^-treated plants after 6 days treatment.

### Measurement and calculation of root parameters

The images were analyzed by RootReader2D (http://www.plantmineralnutrition.net/rootreader. htm), and longest root length (LR) and total root length (TR) were recorded. To evaluate Al^3+^ tolerance for each genotype, root growth parameters were calculated as follows:

Longest root growth (LRG)  =  LR_T6d_ – LR_T0d_*, which represents absolute longest root elongation under Al stress;
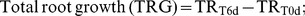


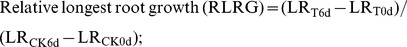


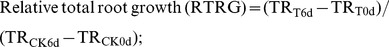



* T and CK represent Al^3+^ treatment and control respectively, and 0d and 6d represent plants with initial and 6 days treatments, respectively.

The correlation analysis among these root parameters was conducted by SPSS (v19.0).

### Population structure, cluster and linkage disequilibrium (LD) analysis

The samples of the whole-genome profiling were analyzed using the Barley PstI (BstNI) version 1.7 array with Diversity Arrays Technology (DArT P/L) in Australia (http://www.triticarte.com.au/content/barley_diversity_analysis.html). A total of 469 barley DArT markers (MAF>0.03) distributed over the whole genome were used to detect population structure using the STRUCTURE software v2.3.3 [39], [40], performing ten independent runs, setting the number of clusters (k) from 1 to 10, with 100,000 MCMC (Markov Chain Monte Carlo) iterations in an admixture model. The largest value of statistic index *Δk*, which was the change rate of LnP(D) (from STRUCTURE output), was used as an indicator of true number of clusters (k) [41]. We calculated genetic distance with NTSYSpc (version 2.10e) and developed a neighbor-joining tree according to Nei [42].

Linkage disequilibrium (LD) between every two linear DArT markers was estimated by TASSEL (v3.0) [43] for Tibetan wild and cultivated barleys individually. The r^2^ values, the squared allele frequency correlations, which is a measurement of the correlation between a pair of variables, and genetic distances were selected to display LD decay curve and fitted equation using Origin Pro (v8.0). LD decay distance in the whole genome was calculated as the genetic distance, when r^2^ = 0.1 using the fitted equation.

### Genome-wide association analysis (GWAS)

Association analysis between 469 DArT markers and four root parameters was performed using three approaches in all accessions (166) using TASSEL (v3.0) [43], [44]. The first approach was Q method, in which Q matrix was included as a cofactor in the regression model to correct population structure. The second approach was k method, and a kinship, which represented the pair-wise relationship matrix, was estimated using software SPAGeDi [44], [45], and considered as a cofactor in the regression model. The third approach was Q+k method, considering both population structure and kinship as cofactors. According to the Q−Q plot from the output of TASSEL, both k and Q+k methods were appropriate for the present study. Then, we conducted GWAS using k method in Tibetan wild and cultivated barleys, respectively. The Benjamini-Hochberg false discovery rate (BH-FDR) [46] of q-value  = 0.01 was applied in association significance test. Manhattan plots were displayed by R software v2.14.2.

### Root index distribution analysis

According to the polymorphism (two type: 1 and 2) of detected markers and subpopulation groups (Wild barley, w; cultivated barley, c) and the root index of accessions were classified into four groups in a box plot. Five detected markers (bpb-6949, bpb-9458, bpb-8524, bpb-0631 and bpb-8021) were analyzed independently. ANOVA analysis was conducted among four groups (w1, w2, c1 and c2) using LSD test.

## Results

### Al tolerance evaluation based on root indexes and their relationships

**Figure 1:**
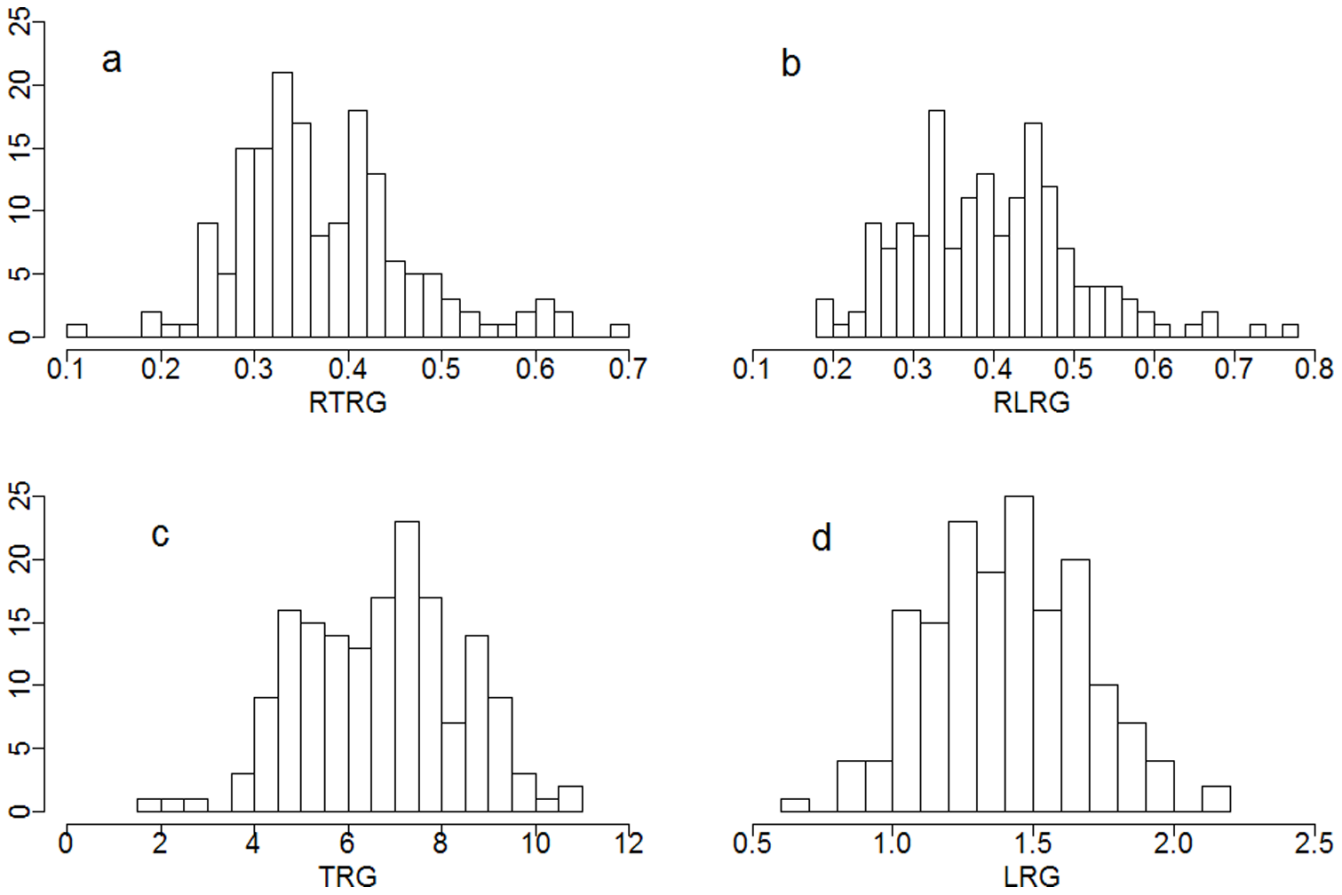
Distribution of four root parameters. Four root parameters were used for evaluation of Al tolerance in 166 accessions. a, RTRG; b, RLRG; c, TRG; d, LRG. The y axis represents frequency of root index.

A total of 110 Tibetan wild accessions and 56 cultivated barleys (Table 1) were evaluated for Al tolerance in response to 5 μM Al^3+^. A wide range of variations in LRG, TRG, RLRG and RTRG were observed (Fig. 1a–d). Under Al^3+^ stress, LRG and TRG were distributed around a mean of 1.40±0.28 (SD) and 6.68±1.70, ranging from 0.64–2.14 and 1.84–10.92, respectively. RLRG and RTRG were present with a mean of 0.373±0.094 and 0.397±0.107, ranging from 0.183–0.767 and 0.116–0.696, respectively. Weak correlations (less than 0.15) were found between root absolute elongation (LRG, TRG) and relative growth (RLRG, RTRG) (Table 1), suggesting that absolute and relative growth could be different in evaluation of barley Al tolerance.

**Table 1:** The correlations between root parameters.

	RTRG*	TRG	RLRG	LRG
RTRG	1.000			
TRG	0.141	1.000		
RLRG	0.776**	−0.005	1.000	
LRG	0.075	0.740**	0.123	1.000

* , RTRG, relative total root growth; TRG, total root growth; RLRG, relative longest root growth; LRG, longest root growth.

** represent significant correlation (P<0.01).

### Genetic division between Tibetan wild and cultivated barleys

**Figure 2:**
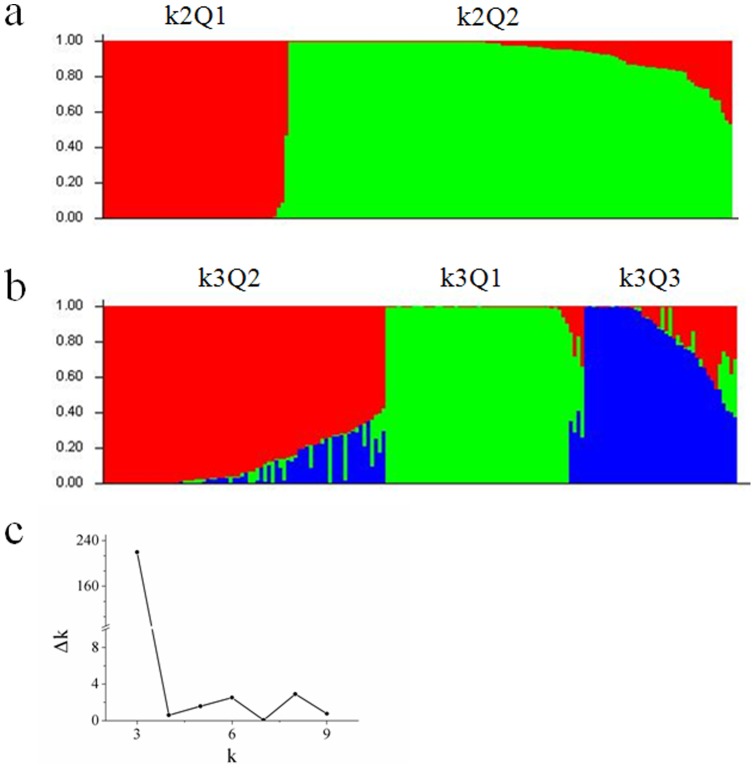
Population structure and Δk. (a, b) Population structure of 166 barley accessions based on genetic diversity detected by 469 DArT markers with k = 2 (a) and k = 3 (b). X axis represents 1–166 accessions. Each accession ordered by membership coefficient (Q) is represented by a line partitioned in colored segments that represent the individual's estimated membership fractions. The number behind k means the total number of subpopulations, and the number behind Q represents which subpopulation the accession belongs to. (c) Δk. Estimation of the most probable number of clusters (k), based on 10 independent runs and k ranging from 1 to 10.

**Figure 3:**
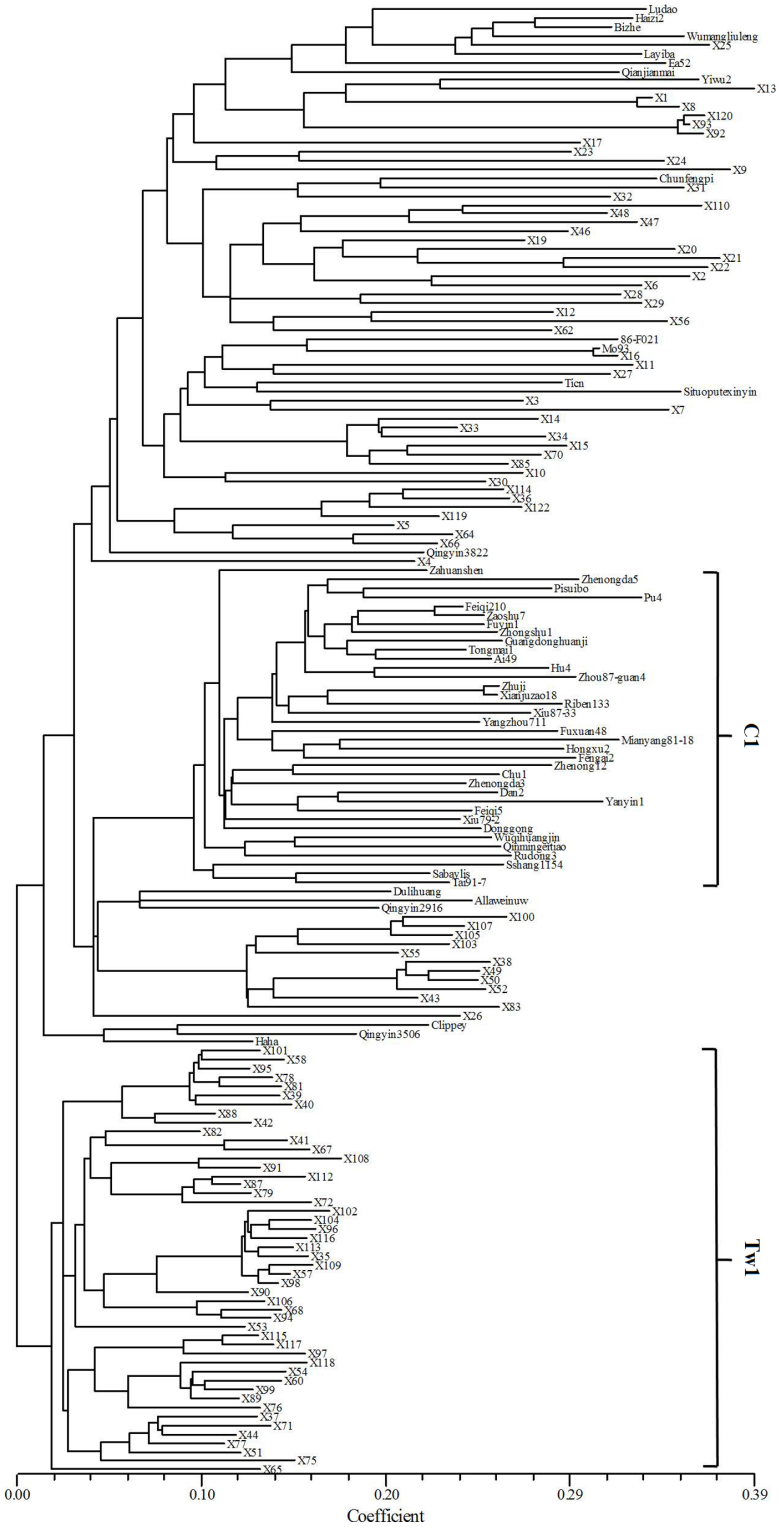
Phylogenetic tree (neighbor-joining) of 166 barley accessions based on 469 DArT markers. Tw1 group consists of 48 Tibetan wild accessions, and C1 group includes 35 cultivated accessions.

Population structure was analyzed using 469 DArT markers. The k2Q1 group, consisting of forty-eight Tibetan accessions with Q value >90%, could be distinctly detected when k was 2 or more (Fig. 2a, b). The result was consistent with the data from the cluster analysis (Fig. 3), in which Tw1 consisting of 48 Tibetan wild accessions showed significant genetic division from the remaining accessions. It is suggested that wide genetic diversity is present in Tibetan wild barley.

To determine the number of subpopulations suitable for association analysis, Δk criterion was applied, and the most evident level of differentiation was observed with k = 3 (Fig. 2c). The k3Q1 consisted of 48 Tibetan accessions, and the same was for k2Q1. Interestingly, k3Q3 (Q>60%) consisted of 32 cultivated barleys (Fig. 2b), revealing the existence of considerable genetic diversity between Tibetan and cultivated barleys. Cluster analysis showed a similar result that 35 cultivated barleys belonged to C1 group (Fig. 3).

**Figure 4:**
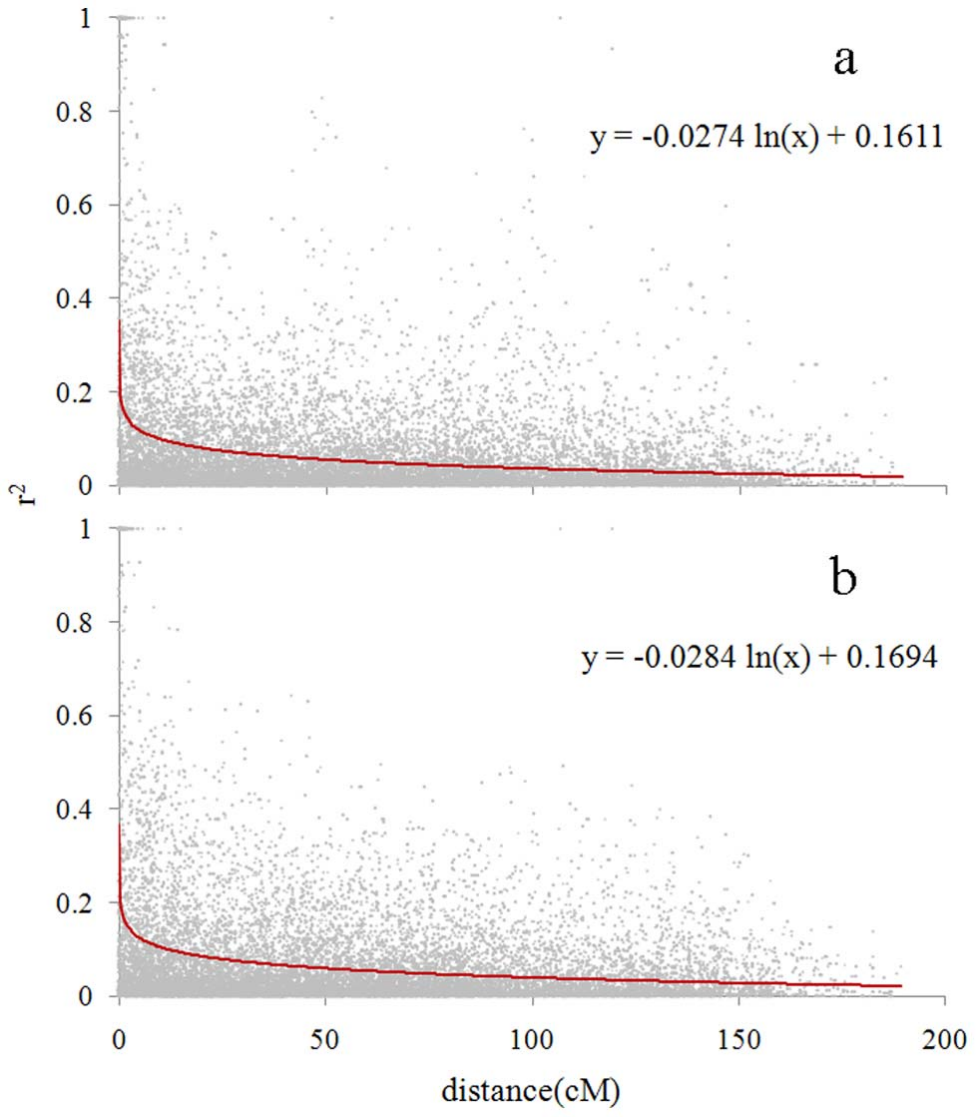
Decay of linkage disequilibrium (LD) of the whole genome of Tibetan wild barleys and cultivated barleys. (a) LD decay in Tibetan wild barleys. (b) LD decay in cultivated barleys. The y axis r^2^ represents the squared allele frequency correlation coefficient between every two linear DArT markers. The decay of genetic distance is 9.30 cM (r^2^ = 0.1) in Tibetan wild barley and 11.52 cM in cultivated barley.

Linkage disequilibrium (LD) decay of genetic distance in the genome of Tibetan and cultivated barleys was 9.30 cM and 11.52 cM (r^2^ = 0.1) (Fig. 4), respectively. Thus 469 DArT markers used in the present study could cover the whole genomic region, and are sufficient for genome wide association analysis.

### Identification of root parameters mediated-Al tolerance loci through GWAS

**Figure 5:**
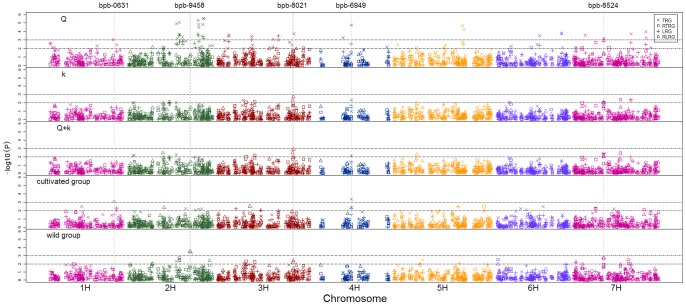
GWA analysis of Al tolerance within and across 56 cultivated and 110 Tibetan wild subgroups. Four root parameters were applied for evaluating Al tolerance: TRG (×), RTRG (□), LRG (+) and RLRG (□;). GWA analysis was firstly conducted using three different methods: Q, k and Q+k methods. Then, k method was selected to perform GWA analysis in cultivated and Tibetan wild subgroups individually. Significant associations were identified using criterion of −log10(P) >2 or 3.

**Figure 6:**
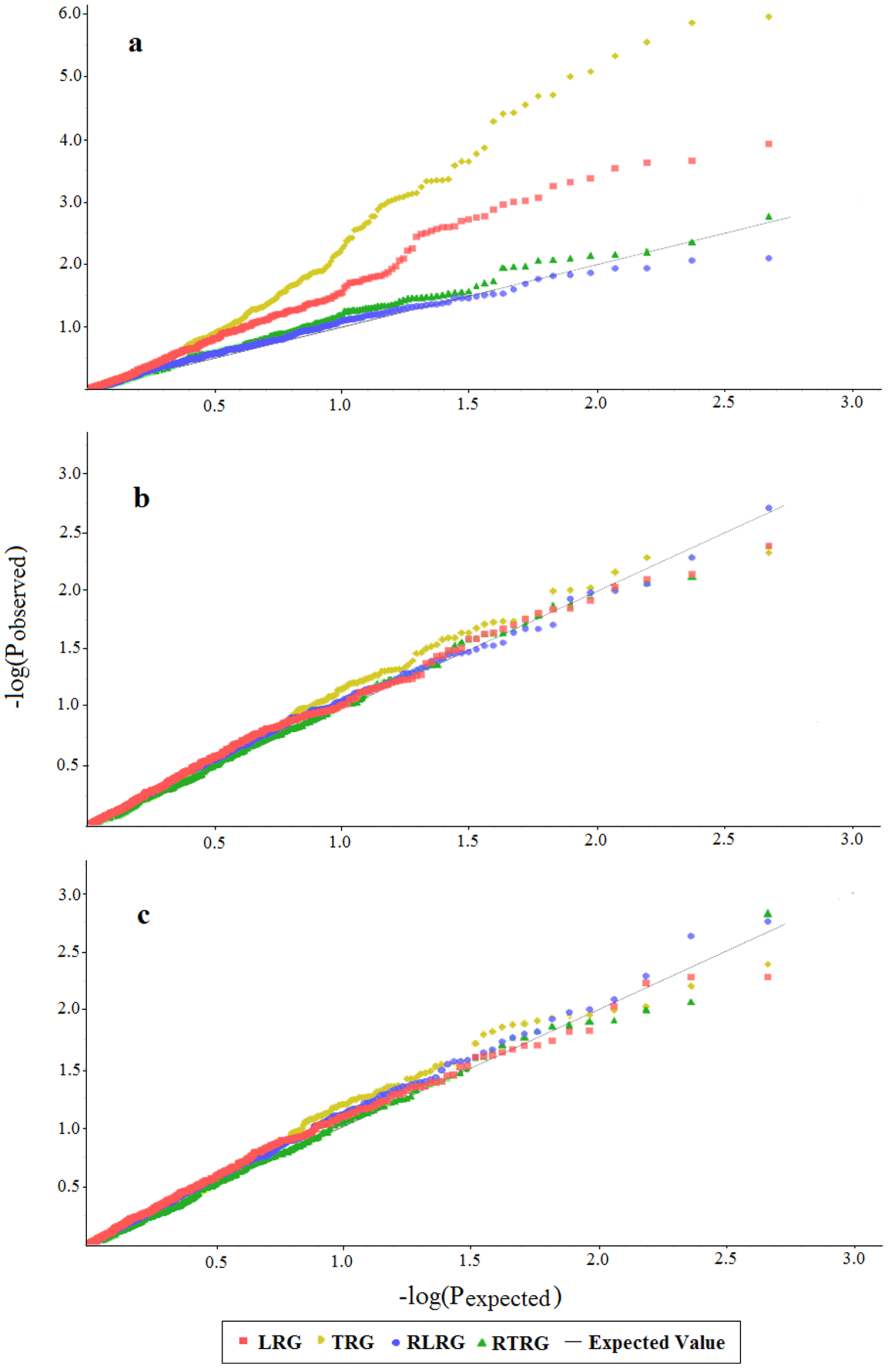
Quantile–quantile (Q-Q) plots of estimated −log_10_(P). Q−Q plots were displayed in four marker-trait association analysis using three models: a Q method, b k method, c Q+k method. The black line is the expected line under the null distribution. Red symbol represents the observed P values for LRG, yellow for TRG, blue for RLRG and green for RTRG.

To identify Al tolerance loci, a genome wide association study was conducted using 469 DArT markers and four root growth parameters (LRG, TRG, RLRG and RTRG) (Fig. 5). When Q method was applied in association analysis across all accessions, 97 marker-trait associations, most of which belonged to TRG and LRG groups, were detected (p<0.01). However, most of these associations were identified as false positive results by Q−Q test (result by TASSEL), because the P values from TRG and LRG groups deviated from the expected value (Fig. 6a). P values from k model and Q+k model were close to expected values, indicating both models are suitable for association analysis (Fig. 6b, c). Eighteen and 16 associations were detected (p<0.01) using the k model and Q+k model, respectively, and 15 associations detected using both models.

Association analysis was also performed in subgroups. Twenty-seven and 24 associations were detected in the cultivated and wild groups, respectively (p<0.01). When the threshold of significant test was set at p<0.001, only three regions were detected. In the cultivated group, bpb-6949 on Chr.4H and bpb-0631 on Chr.1H explained 25.6% and 23.1% of the phenotypic variation, respectively. In the wild group, bpb-9458 and bpb-0590 on Chr.2H both explained 12.9% of the phenotypic variation (p<0.001). It was worth mentioning that three root parameters (LRG, RLRG and TRG) were associated with bpb-8524 and bpb-6707 on Chr.7H, suggesting these markers were powerfully correlated with Al tolerance, and explaining 9.7% and 9.5% of phenotypic variation. Bpb-8021 on Chr.3H was detected as the most significant marker across all accessions using both k and Q+k methods, but certified as false positive association by following distribution analysis.

### Root index distribution of the detected five markers

**Figure 7:**
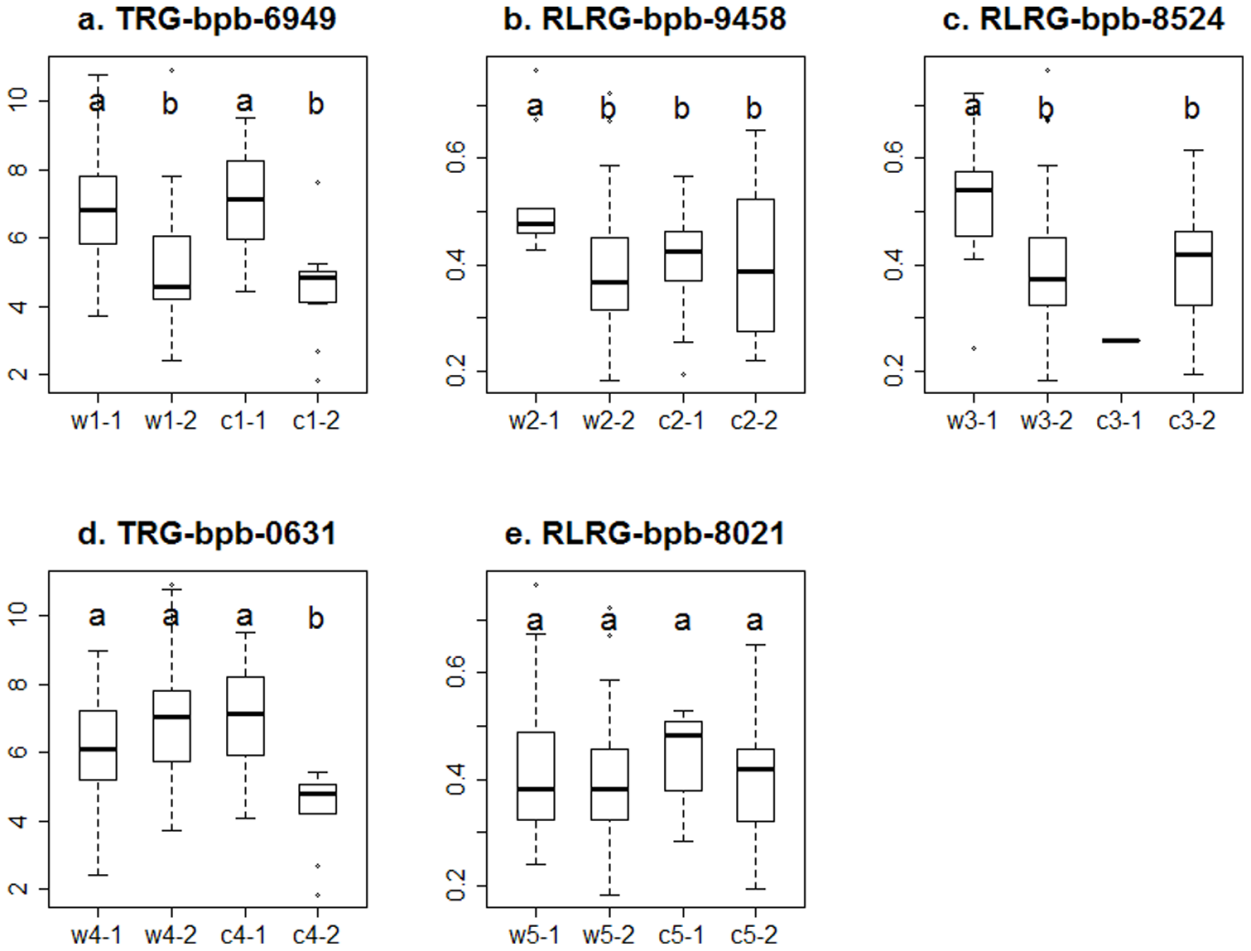
Distribution of root index bases on marker polymorphism of five markers in Tibetan wild and cultivated subgroups. For each marker, four groups were included: w n-1, w n-2, c n-1 and c n-2, in which w represents Tibetan wild group, c represents cultivated group, 1 and 2 are DArT marker polumorphism, n = 1–5 represents five different DArT markers. The markers and root indexes include: (a) marker bpb-6949 associated with TRG, (b) bpb-9458with RLRG, (c) bpb-8524with RLRG, (d) bpb-0631with TRG, (e) bpb-8021with RLRG. For each marker, ANOVA analysis was conducted among four groups, and significant difference was marked by different letters.

Based on marker polymorphisms, the distribution of root index were examined within cultivated and Tibetan barleys based on markers bpb-6949, bpb-9458, bpb-8524, bpb-0631 and bpb-8021 (Fig. 7a–e). W1-1 (95 accessions) showed higher TRG than w1-2 (15 accessions), which was similar to the result in the cultivated group (Fig. 7a). However, bpb-6949 was detected in all accessions and cultivated group by GWA, but not in the wild group. It may be explained that this Al^3+^-tolerant locus commonly existed in barley accessions, but its effect may be reduced or covered by other loci in the Tibetan group due to abundant genetic diversity.

W2-1 (10 accessions) and w3-1 (7 accessions) showed higher RLRG than that the other three groups, whereas no significant difference in RLRG was found between groups in the cultivated group (Fig. 7b, c). Moreover, only one accession was presented in haplotype c3-1 with low RLRG. These results confirmed that bpb-9458 and bpb-8524 associated with Al^3+^-tolerant genes were Tibetan group-specific.

C4-2 showed lower TRG than the other three groups, indicating bpb-0631 was cultivated group-specific, which was consistent with the results obtained by GWA analysis (Fig. 7d). Although bpb-8021 showed the most significance in GWA analysis across all accessions, no significant difference was found among groups within this marker, suggesting that it is a false positive association (Fig. 7e).

## Discussion

### GWAS results are affected by phenotyping methods

Many researchers usually consider relative longest root growth as an indicator for Al tolerance evaluation [3]–[7]. However, it was reported that the application of different phenotypic indexes directly impacted the significance of QTLs detected in rice Al tolerance [13], which was confirmed in the current study, that different loci were detected as associated with Al tolerance by using different root parameters in GWAS. Although there were significant corrections between LRG and TRG, and between RLRG and RTRG, most of loci detected are different.

Therefore, the question may be raised as to which index could be recognized as reasonable indicators for Al tolerance evaluation. In the present study, different subpopulations needed different root parameters to identify Al tolerance loci. When absolute root growth (LRG, TRG) indexes and relative root growth (RLRG, RTRG) indexes were used, 22 and 5 loci were detected respectively in the cultivated group (Table 2, Fig. 5). The different results were obtained in the Tibetan group, with 6 loci being detected using absolute root growth (LRG, TRG) indexes and 18 loci using relative root growth (RLRG, RTRG) indexes. The number of detected loci showed that absolute root growth (LRG, TRG) indexes are more suitable for the cultivated group, and relative root growth (RLRG, RTRG) indexes for the Tibetan group. However, the underlying reason is not known. Furthermore, the most significant locus bpb-6949 in the cultivated group was associated with TRG index, while bpb-9458 was detected using RLRG index in the Tibetan group. This result suggests that Al tolerance strategies differ among barley subpopulations and is mediated by developmental types of roots.

**Table 2:** Lists of DArT markers with a significant marker-trait association using different model (k and Q+k methods) and within subgroups.

	Trait	Marker	Chr.	Distance(cM)	Frequency(%)	−log_10_(p)	r^2^
k	LRG	bPb-9754	2H	82.77	13.9	2.04	0.045
	LRG	bPb-6706	7H	58.17	4.8	2.15	0.046
	LRG	bPb-8524	7H	58.02	4.8	2.10	0.044
	LRG	bPb-9908	7H	111.69	24.1	2.38	0.052
	RLRG	bPb-1072	2H	70.03	44.6	2.00	0.042
	RLRG	bPb-3907	3H	146.78	16.3	2.29	0.049
	RLRG	bPb-8021	3H	147.95	16.3	2.71	0.060
	RLRG	bPb-8524	7H	58.02	4.8	2.06	0.045
	TRG	bPb-0858	2H	95.68	39.2	2.03	0.043
	TRG	bPb-4040	2H	82.13	15.7	2.16	0.046
	TRG	bPb-7024	2H	12.92	5.4	2.01	0.041
	TRG	bPb-4660	3H	50.43	45.2	2.29	0.051
	TRG	bPb-6949	4H	72.21	16.3	2.33	0.050
	TRG	bPb-9908	7H	111.69	24.1	2.30	0.050
	RTRG	bPb-3805	3H	72.18	29.5	2.13	0.049
	RTRG	bPb-6347	3H	55.63	25.3	2.07	0.046
	RTRG	bPb-8492	6H	26.51	46.4	2.03	0.042
	RTRG	bPb-2748	7H	91.26	22.9	2.39	0.057
Q+k	LRG	bPb-6706	7H	58.17	4.8	2.42	0.053
	LRG	bPb-8524	7H	58.02	4.8	2.36	0.052
	RLRG	bPb-1072	2H	70.03	44.6	2.45	0.055
	RLRG	bPb-3907	3H	146.78	16.3	2.45	0.054
	RLRG	bPb-8021	3H	147.95	16.3	2.88	0.066
	RLRG	bPb-3792	5H	45.58	22.3	2.27	0.049
	RLRG	bPb-8524	7H	58.02	4.8	2.07	0.045
	TRG	bPb-0615	2H	11.28	5.4	2.11	0.042
	TRG	bPb-7024	2H	12.92	5.4	2.15	0.043
	TRG	bPb-0858	2H	95.68	39.2	2.25	0.046
	TRG	bPb-6949	4H	72.21	16.3	2.11	0.042
	TRG	bPb-6706	7H	58.17	4.8	2.35	0.049
	TRG	bPb-8524	7H	58.02	4.8	2.25	0.046
	RTRG	bPb-3805	3H	72.18	29.5	2.19	0.051
	RTRG	bPb-6347	3H	55.63	25.3	2.01	0.045
	RTRG	bPb-2748	7H	91.26	22.9	2.42	0.058
cultivated group	LRG	bPb-1959	1H	133.12	14.3	2.33	0.158
	LRG	bPb-2203	3H	35.93	7.1	2.21	0.162
	LRG	bPb-0353	3H	84.38	7.1	2.04	0.133
	LRG	bPb-8015	3H	84.89	7.1	2.04	0.133
	LRG	bPb-6949	4H	72.21	21.4	2.31	0.157
	LRG	bPb-4758	5H	126.53	14.3	2.49	0.173
	LRG	bPb-0071	5H	126.77	14.3	2.45	0.171
	LRG	bPb-9601	7H	42.67	3.8	2.37	0.162
	RLRG	bPb-8700	2H	72.09	6.6	2.38	0.162
	RLRG	bPb-1012	3H	62.86	28.6	2.54	0.210
	TRG	bPb-0631	1H	128.45	17.9	3.11	0.231
	TRG	bPb-5290	1H	64.89	30.4	2.51	0.175
	TRG	bPb-1959	1H	133.12	14.3	2.00	0.130
	TRG	bPb-6822	2H	114.40	12.5	2.24	0.151
	TRG	bPb-6087	2H	145.55	37.5	2.18	0.147
	TRG	bPb-1066	2H	139.75	37.5	2.14	0.143
	TRG	bPb-0326	2H	139.91	3.6	2.14	0.144
	TRG	bPb-3536	2H	132.78	26.8	2.06	0.148
	TRG	bPb-6949	4H	72.21	21.4	3.36	0.256
	TRG	bPb-7987	4H	72.27	16.1	2.39	0.164
	TRG	bPb-0432	6H	91.99	32.1	2.49	0.176
	TRG	bPb-0934	6H	47.81	17.9	2.25	0.155
	TRG	bPb-7179	6H	58.56	16.1	2.12	0.140
	TRG	bPb-1447	7H	78.22	3.6	2.20	0.147
	RTRG	bPb-5413	5H	177.26	8.9	2.48	0.175
	RTRG	bPb-9601	7H	42.67	3.8	2.23	0.150
	RTRG	bPb-1793	7H	137.20	3.6	2.12	0.143
Tibetan group	LRG	bPb-4494	5H	127.88	3.6	2.05	0.065
	LRG	bPb-7559	7H	3.02	19.1	2.21	0.072
	LRG	bPb-0844	7H	5.01	19.1	2.21	0.072
	RLRG	bPb-9458	2H	122.28	9.1	3.55	0.129
	RLRG	bPb-0590	2H	123.70	9.1	3.43	0.129
	RLRG	bPb-6048	2H	161.12	14.5	2.41	0.080
	RLRG	bPb-5629	2H	49.03	10.9	2.03	0.065
	RLRG	bPb-8021	3H	147.95	20.9	2.36	0.078
	RLRG	bPb-3907	3H	146.78	20.0	2.19	0.071
	RLRG	bPb-6710	5H	51.63	23.6	2.02	0.066
	RLRG	bPb-8524	7H	58.02	6.4	2.78	0.097
	RLRG	bPb-6706	7H	58.17	6.4	2.70	0.095
	RLRG	bPb-0202	7H	106.63	17.3	2.53	0.087
	TRG	bPb-0858	2H	95.68	46.4	2.31	0.077
	TRG	bPb-6260	5H	56.76	42.7	2.48	0.084
	TRG	bPb-9908	7H	111.69	22.7	2.07	0.066
	RTRG	bPb-9423	1H	48.95	19.1	2.00	0.067
	RTRG	bPb-7991	2H	101.27	30.0	2.72	0.093
	RTRG	bPb-1926	2H	102.12	30.9	2.49	0.083
	RTRG	bPb-6194	2H	102.38	30.0	2.44	0.081
	RTRG	bPb-6347	3H	55.63	16.4	2.30	0.085
	RTRG	bPb-3805	3H	72.18	30.9	2.25	0.084
	RTRG	bPb-3780	6H	3.55	47.3	2.54	0.086
	RTRG	bPb-0202	7H	106.63	17.3	2.05	0.067

### Tibetan wild barley provides elite germplasm for barley improvement in stress tolerance

Genetic differentiation is present between Tibetan wild and cultivated barleys. This is well supported by population structure partition (k = 2) and the genetic division of Tw1 group in cluster analysis in the current study (Fig. 2a and 3). Therefore, the Tibetan wild barley could provide elite germplasm for barley improvement in abiotic and biotic stress tolerance to fight against unpredictable climate changes in the future.

Structure analysis and Δk showed that three estimated populations were present in all 166 accessions (Fig. 2b, c). A subpopulation k3Q3 group consisting of 32 cultivated cultivars, was segregated from k2Q2 group. It may be suggested that cultivated barley gradually developed and engendered its own novel physiological or molecular traits or Al tolerant strategies during its expansion into specific environments. A good example is that an Al-resistant cultivar Murasakimochi acquires enhanced Al tolerance by a 1 kb insertion in the upstream region of *HvAACT1* [47]. This insertion happened during the expansion of barley cultivation onto acid soil area and was identified to be originated from Eastern Asian region [47]. However, there is no such insertion in all wild barley accessions used in the present study (Data not shown).

LD decay analysis showed that DArT markers used in this study could cover four times as the size of the barley genome, indicating the density of DArT markers sufficient for GWAS. LD decayed more rapidly in Tibetan barley (9.30 cM) than cultivated barley (11.52 cM), thus the results of GWAS in Tibetan wild group can provide higher resolution of fine mapping and gene discovery than those in cultivated barley.

In short, it may be concluded that Tibetan wild barley is appropriate and efficient for genome-wide association by which we may detect rare elite alleles contributing to elevated Al tolerance in barley.

### Novel Al-tolerant loci are detected by GWAS

GWA mapping by using 110 Tibetan wild and 56 cultivated barleys genotyped with 469 DArT markers identified four significant regions, all of which are subpopulation-specific.

In the cultivated group, bpb-6949 was localized at 0.8 cM away from a major QTL in Chr.4H and a candidate gene *HvMATE*, which is considered as a major gene conferring barley Al tolerance [26], [48]. This marker was also detected (p<0.01) when all barley genotypes were examined, suggesting that it is a common locus related to Al tolerance in barley.

However, the genetic diversity of tolerant genotypes used in a previous QTL study was limited [49]–[53]. Only the alleles segregating between parents of DH population could be identified by the QTL analysis. *HvMATE (HvAACT1)* was identified by using Dayton and Murasakimochi [4], [26]. The two genotypes, as well as WB229 and FM404 were used as tolerant parents in the previous QTL studies. All are cultivated barleys. In the present study, GWAS was also performed in the Tibetan group and novel loci were identified on Chr.2H and Chr.7H. This result strongly suggests that different Al tolerant mechanism exists in Tibetan barley. The genotypes selected from haplotype analysis and the detected loci could be used in marker-assistant breeding. However, these possible Al-tolerant genes are still unknown, and fine mapping is required for gene discovery in future research.
